# Cofactor engineering through heterologous expression of an NADH oxidase and its impact on metabolic flux redistribution in *Klebsiella pneumoniae*

**DOI:** 10.1186/1754-6834-6-7

**Published:** 2013-01-25

**Authors:** Xiao-Jun Ji, Zhi-Fang Xia, Ning-Hua Fu, Zhi-Kui Nie, Meng-Qiu Shen, Qian-Qian Tian, He Huang

**Affiliations:** 1 State Key Laboratory of Materials-Oriented Chemical Engineering, College of Biotechnology and Pharmaceutical Engineering, Nanjing University of Technology, No. 5 Xinmofan Road, Nanjing, 210009, People’s Republic of China

**Keywords:** Acetoin, 2,3-Butanediol, Cofactor engineering, *Klebsiella pneumoniae*, NADH oxidase

## Abstract

**Background:**

Acetoin is an important bio-based platform chemical. However, it is usually existed as a minor byproduct of 2,3-butanediol fermentation in bacteria.

**Results:**

The present study reports introducing an exogenous NAD^+^ regeneration sysytem into a 2,3-butanediol producing strain *Klebsiella pneumoniae* to increse the accumulation of acetoin. Batch fermentation suggested that heterologous expression of the NADH oxidase in *K. pneumoniae* resulted in large decreases in the intracellular NADH concentration (1.4 fold) and NADH/NAD^+^ ratio (2.0 fold). Metabolic flux analysis revealed that fluxes to acetoin and acetic acid were enhanced, whereas, production of lactic acid and ethanol were decreased, with the accumualation of 2,3-butanediol nearly unaltered. By fed-batch culture of the recombinant, the highest reported acetoin production level (25.9 g/L) by *Klebsiella* species was obtained.

**Conclusions:**

The present study indicates that microbial production of acetoin could be improved by decreasing the intracellular NADH/NAD^+^ ratio in *K. pneumoniae*. It demonstrated that the cofactor engineering method, which is by manipulating the level of intracellular cofactors to redirect cellular metabolism, could be employed to achieve a high efficiency of producing the NAD^+^-dependent microbial metabolite.

## Background

Metabolic engineering has been widely applied in modifying metabolic pathways to improve the properties of microbial strains, including manipulation of enzyme levels through the amplification, disruption, or addition of a metabolic pathway [1]. These approaches have been proven to be powerful in developing microbial strains for the commercial production of organic acids, amino acids, biofuels, and pharmaceuticals [2]. Nevertheless, over-expression, deletion, or introduction of heterologous genes in target metabolic pathways do not always result in the desired phenotype [3]. In recent years, cofactor engineering, which is considered as a new branch of metabolic engneering, has attracted increasing attention. Manipulations of the cofactor form and level have become a useful tool for metabolic engineering to redistribute/enhance carbon flux in metabolic networks [4]. Nicotinamide adenine dinucleotides (NADH and NAD^+^), as one pair of key cofactors play an important role in over 300 biochemical reactions involving oxidation and reduction [2,5]. Therefore, this cofactor pair (NADH and NAD^+^) has a critical effect on maintaining the intracellular redox balance, which is a basic condition for microorganism to metabolize and grow [6]. Regulation of the NADH/NAD^+^ ratio can be achieved by either weakening the metabolic pathways competing for NADH or NAD^+^[7-9], or introducing an NADH or NAD^+^ regeneration system. Intracellular concentrations of NADH and NAD^+^ can be changed by expressing an NAD^+^-dependent formate dehydrogenase (EC 1.2.1.2; FDH), an NADH oxidase (EC 1.6.99.3; NOX), or a nicotinic acid phosphoribosyl transferase (EC 2.4.2.11; NAPRTase). Increase of intracellular NADH availability by overexpressing FDH in bacteria provoked a significant metabolic redistribution [10,11]. Heterologous expression of NOX was conducted in *Lactococcus latis*, resulting in a low NADH/NAD^+^ ratio and the shift from homolactic fermentation to mixed-acid fermentation under aerobic conditions [4]. Overexpressing the gene of *pnc*B encoding NAPRTase in *E. coli*, was observed to increase the total NAD^+^ level and decrease the NADH/NAD^+^ ratio [12]. These studies showed that the expression of the NADH or NAD^+^-related enzymes could lead to a dramatically altered NADH/NAD^+^ ratio and a significantly changed spectrum of metabolic products [11].

Cofactor engineering has been successfully applied for the prodcution of many bio-based chemicals, such as 1,3-propanediol [9,11], pyruvic acid [13], and succinic acid [14]. In the present work, this novel and powerful technology will be applied to the production of another important bio-based chemical, acetoin. Acetoin is defined as one of the high value-added platform compounds and selected by the U. S. Department of Energy as one of the potential top 30 chemical building blocks from sugars [15]. A number of microorganisms are able to accumulate acetoin, including the genera *Klebsiella*, *Paenibacillus*, *Bacillus*, *Serratia*, etc. [16,17]. However, these strains are widely known as good producers of 2,3-butanediol (2,3-BD) which is another important bio-based platform chemical [18]. Acetoin is only generated as a minor by-product. Although some *Bacillus* strains have been used for acetoin production [19-21], the long fermentation period generally needed in fermentative acetoin using *Bacillus* strains still hinder its large-scale production.

In bacteria, acetoin and 2,3-BD are produced by the mixed acid-2,3-BD fermentation pathway. In the presence of NAD^+^, 2,3-BD dehydrogenase (EC 1.1.1.76; BDH), which is a NADH-dependent dehydrogenase, can catalyze 2,3-BD to acetoin [22] (Additional file 1: Figure S1). Due to the reversible transformation between acetoin and 2,3-BD coupled with the NADH/NAD^+^ conversion, the synthesis of these two products have been considered involving in regulation of the NADH/NAD^+^ ratio in bacteria [23]. That is to say, these two products exist in the same branch of the metabolic pathway linked with NADH and NAD^+^ transformation. Considering that *Klebsiella* species are the most powerful 2,3-BD producers, they could be engineered to obtain high acetoin producing ability. In this work, an NAD^+^ regeneration system (*nox-2* gene from *Streptococcus pneumoniae*, encoding NOX) was introduced into a 2,3-BD producing strain *Klebsilla pneumoniae* to manipulate the intracellular NAD^+^ level and NADH/NAD^+^ ratio for acetoin overproduction. The consequent effect on the distribution of metabolites in *K. pneumoniae*, especially, on the production of compounds that require NADH or NAD^+^ for their synthesis, including acetoin, 2,3-BD, and other by-products, such as ethanol, lactic acid, and acetic acid, was investigated.

## Results

### Expression of the *S. pneumoniae nox*-2 gene in *K. pneumoniae* and its effect on cell growth

**Figure 1:**
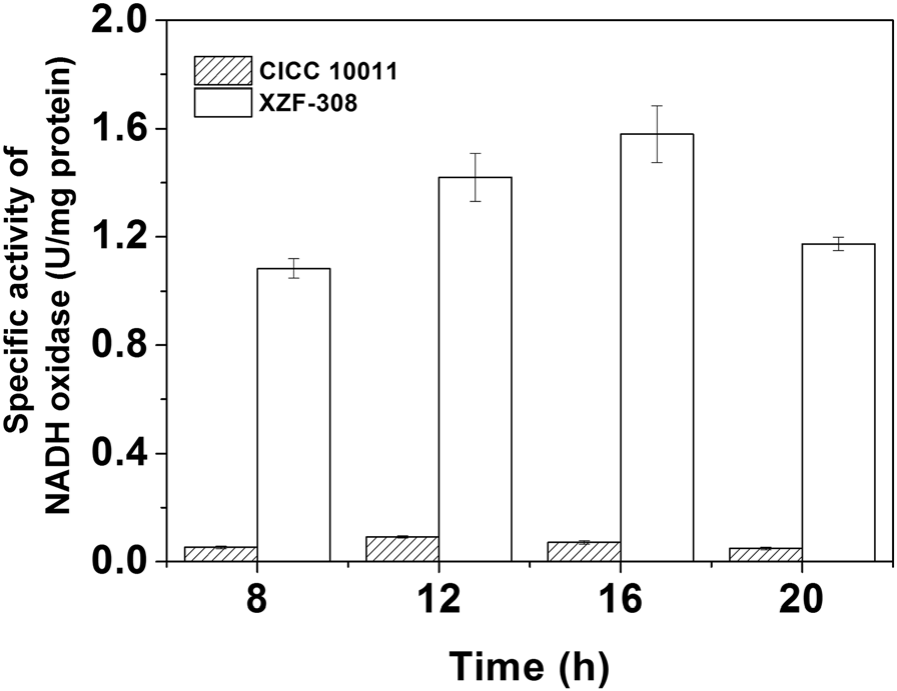
**Time courses of NADH oxidase activity of the parent and the recombinant *Klebsiella pneumoniae*.** CICC 10011: the parent strain; XZF-308: the recombinant strain.

To investigate the effect of NAD^+^ regeneration on acetoin production, the *nox-2* gene from *S. pneumoniae* encoding the water-forming NOX was cloned and overexpressed in a 2,3-BD producing strain *K. pneumoniae*. As indicated in the section of ‘Materials and methods’, the confirmed recombinant XZF-308 was obtained. The NOX activities of the recombinant and the parent strain were determined. As shown in Figure 1, the highest specific activity of NOX (1.57 U/mg protein) was found in the recombinant strain when cells entered exponential growth phase. While NOX activity in the parent strain was extremely low during the entire fermentation period, revealing that NOX was positively produced in the recombinant strain (Additional file 2: Figure S2).

**Figure 2:**
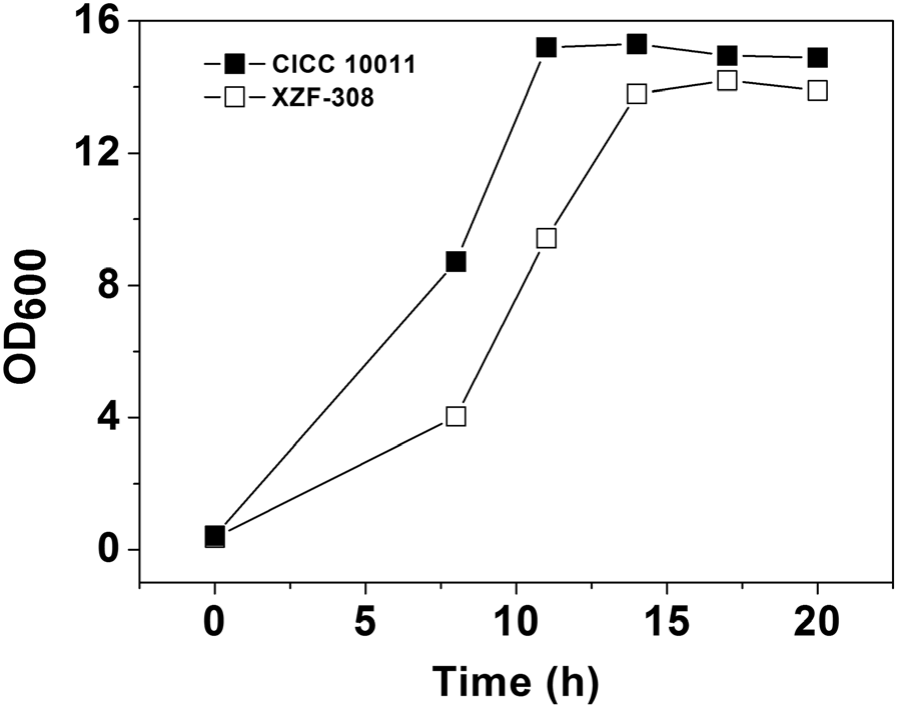
**Growth curves of the parent and the recombinant *Klebsiella pneumoniae.*** CICC 10011: the parent strain; XZF-308: the recombinant strain.

To investigate the effect of NOX overexpression on cell growth, the recombinant strain and the parent strain were cultured at the same conditions. As shown in Figure 2, the recombinant strain grew to a lower OD_600_ as compared to the parent strain, indicating that the cell growth of *K. pneumoniae* was inhibited due to the heterologous expression of NOX. At the first 8 h, the cell growth rate of the recombinant strain (0.18 g/(L·h)) reached only around half of that the parent strain achieved (0.38 g/(L·h)). The growth rates for both strains were then increased (8–11 h) but the growth rate of the recombinant strain (0.63 g/(L·h)) was still lower compared to the parent strain (0.76 g/(L·h)). At the stage of 11–14 h, although the recombinant strain grew faster than the parent strain, its biomass accumulation was still lower than the parent strain, and this trend lasted to the end.

### Effect of the heterologous NOX expression on intracellular concentrations of NADH and NAD^+^

**Figure 3:**
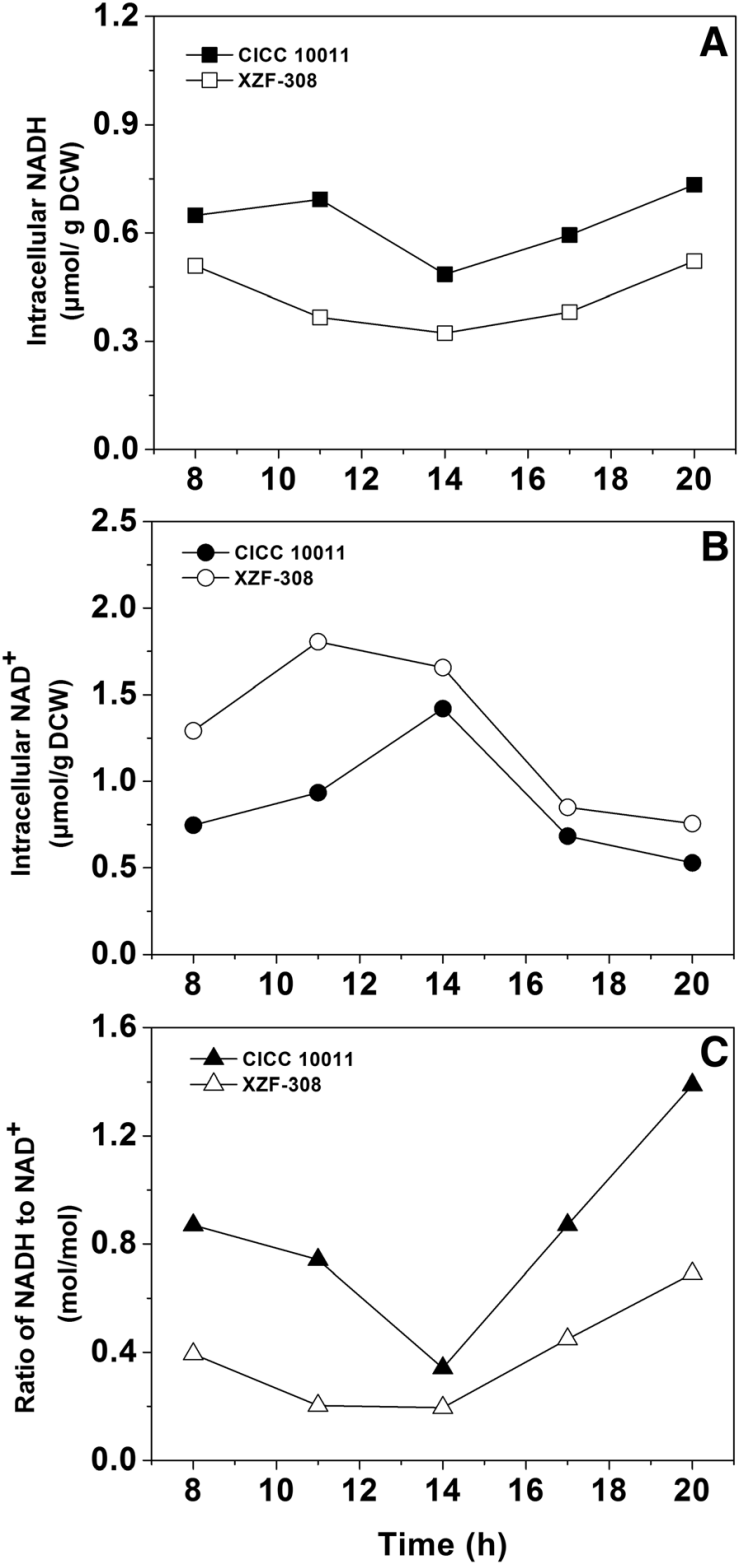
**Effects of the NAD**^**+**^**regeneration system introduced into *Klebsiella pneumoniae *on the concentrations of intracellular NADH, NAD**^**+**^**, and NADH/NAD**^**+**^**ratio in batch fermentation.** CICC 10011: the parent strain; XZF-308: the recombinant strain.

Heterologous expression of NOX in *K. pneumoniae* was expected to decrease the total intracellular NADH pool and thus strengthen the flux of NAD^+^-dependent pathways. As shown in Figure 3A and B, for both the parent and recombinant strains, the intracellular contents of NADH and NAD^+^ were constantly changing with the fermentation time. During the growth stage (0–14 h), the NADH concentration in the parent strain was increasing firstly and then decreasing, which was different from the declining trend in the recombinant strain. While the NAD^+^ levels in both strains were increasing continuously in the whole stage. However, when the cells entered into the non-growth stage (after 14 h), the NADH concentration in both strains was increasing and NAD^+^ concentration was decreasing. Furthermore, heterologous expression of NOX in *K. pneumoniae* led to a lower level of NADH pool and a higher level of NAD^+^ pool when compared to the parent strain. The difference of the NADH/NAD^+^ ratio between the parent and the recombinant strain was also observed (Figure 3C). In fed-batch fermentation, this difference was still existed (Additional file 3: Figure S3). This indicated that the redox status in *K. pneumoniae* was disturbed, which explained why cell growth of the recombinant was negatively affected (Figure 2).

### Introduction of NAD^+^ regeneration system leads to metabolic flux redistribution in *K. pneumoniae*

Metabolite profiles of the parent and the recombinant strains were monitored in parallel batch cultures. As described in the first part of ‘Results’ section, the recombinant strain positively expressed NOX, which helped regenerate the intracellular NAD^+^ from NADH. Therefore, the carbon fluxes of the NADH-dependent pathways were expected to be triggered to decrease in the recombinant strain. In the mixed acid-2,3-BD fermentation pathway of *K. pneumoniae* (major products: acetoin and 2,3-BD), the by-products included acetic acid, lactic acid, and ethanol, among which lactic acid and ethanol formation competed with 2,3-BD for NADH [22].

**Figure 4:**
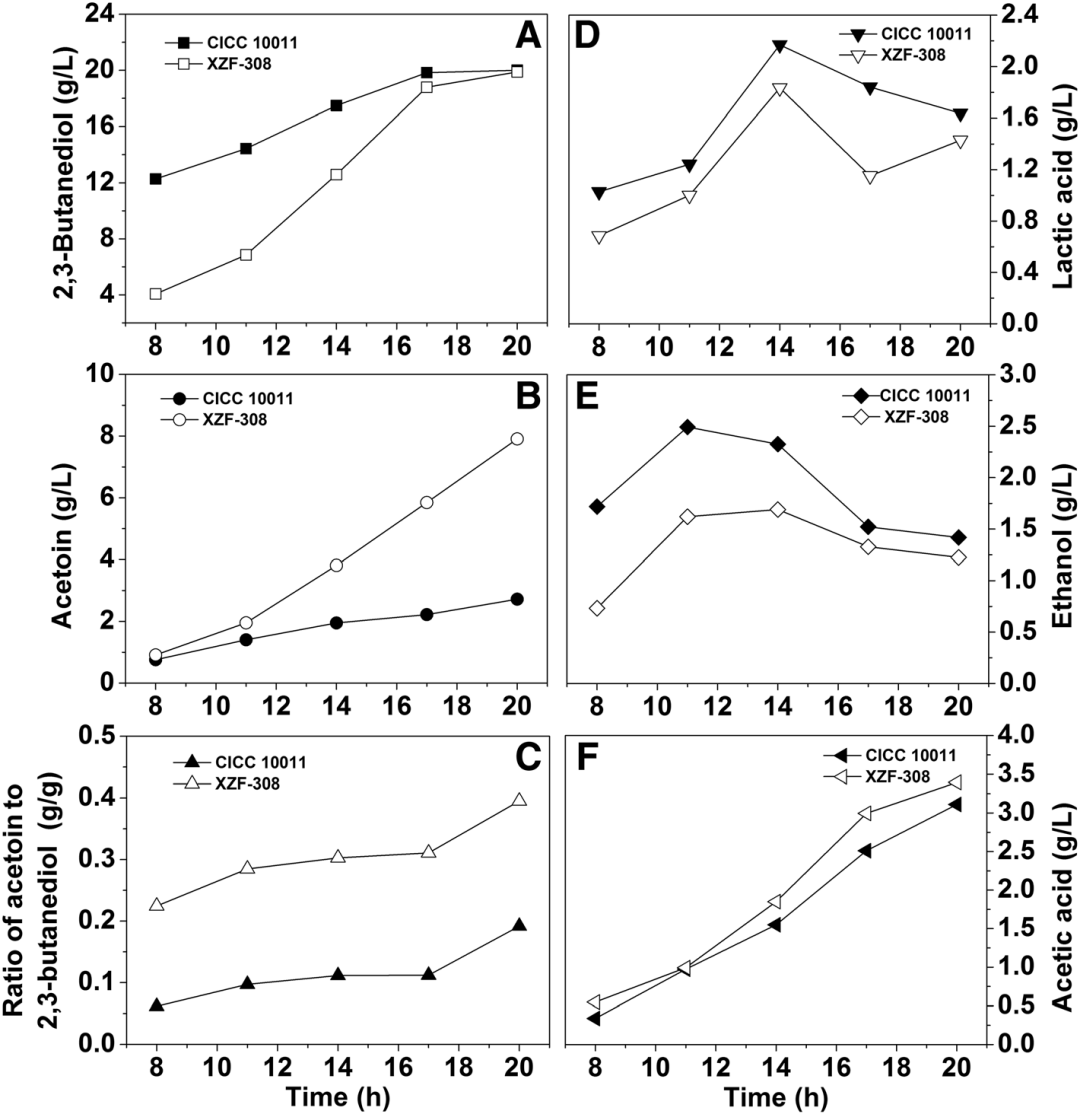
**Effects of the NAD**^**+**^**regeneration system introduced into *Klebsiella pneumoniae *on the metabolite distribution in batch culture.** CICC 10011: the parent strain; XZF-308: the recombinant strain.

To investigate the metabolic changes of *K. pneumoniae* in response to the heterologous expression of NOX, the concentrations of major metabolites of the recombinant and the parent strains were determined (Figure 4). The recombinant strain produced more acetoin and acetic acid, less ethanol and lactic acid compared to the parent strain probably due to the result of decreased NADH availability. Molar yields of 2,3-BD, lactic acid, and ethanol were 0.5%, 12.2%, and 12.9% lower than those of the parent strain, respectively, while molar yields of acetoin and acetic acid were 189.29% and 9.5% higher (Table 1). This suggested that the cellular carbon fluxes were redistributed.

**Table 1:** **Effect of the NAD**^**+**^**regeneration system introduction on the metabolic flux distribution in *Klebsiella pneumoniae *(unit: mol/mol glucose)**

**Metabolites**^**a**^	***K. pneumoniae *CICC10011**	***K. pneumoniae *XZF-308**	**Fluxes redistributed by introducing the NAD**^**+**^**regeneration system**
Acetoin	0.056	0.162	189.29%
2,3-Butanediol	0.400	0.398	−0.5% ^b^
Acetic acid	0.093	0.102	9.5%
Lactic acid	0.033	0.029	−12.2%^b^
Ethanol	0.055	0.048	−12.9% ^b^

^a^ Acetoin is an NAD^+^ dependent metabolite, 2,3-butanediol, lactic acid, and ethanol are NADH dependent metabolites, while acetic acid is not dependent of NADH or NAD^+^.

^b^ Negative standard means a decrease in glucose flux to 2,3-butanediol, lactic acid, and ethanol.

Due to the alteration of intracellular NADH and NAD^+^ pools, the accumulations of acetoin, 2,3-BD, acetic acid, lactic acid, and ethanol in the fermentation broth of the recombinant strain were different from the parent strain. During the fermentation process, acetoin were produced at higher rates by the recombinant strain (Figure 4B), and the final yields of acetoin and acetic acid were also higher compared to the parent strain. However, the final yield of 2,3-BD was similar for both strains, while ethanol and lactic acid production of the parent strain was less. This suggested that the NADH-dependent pathways, including the pathways from pyruvic acid to ethanol, lactic acid, and 2,3-BD, reacted differently in response to the introduction of NAD^+^ regeneration system*.*

### Production of acetoin by fed-batch culture of the recombinant *K. pneumoniae*

**Figure 5:**
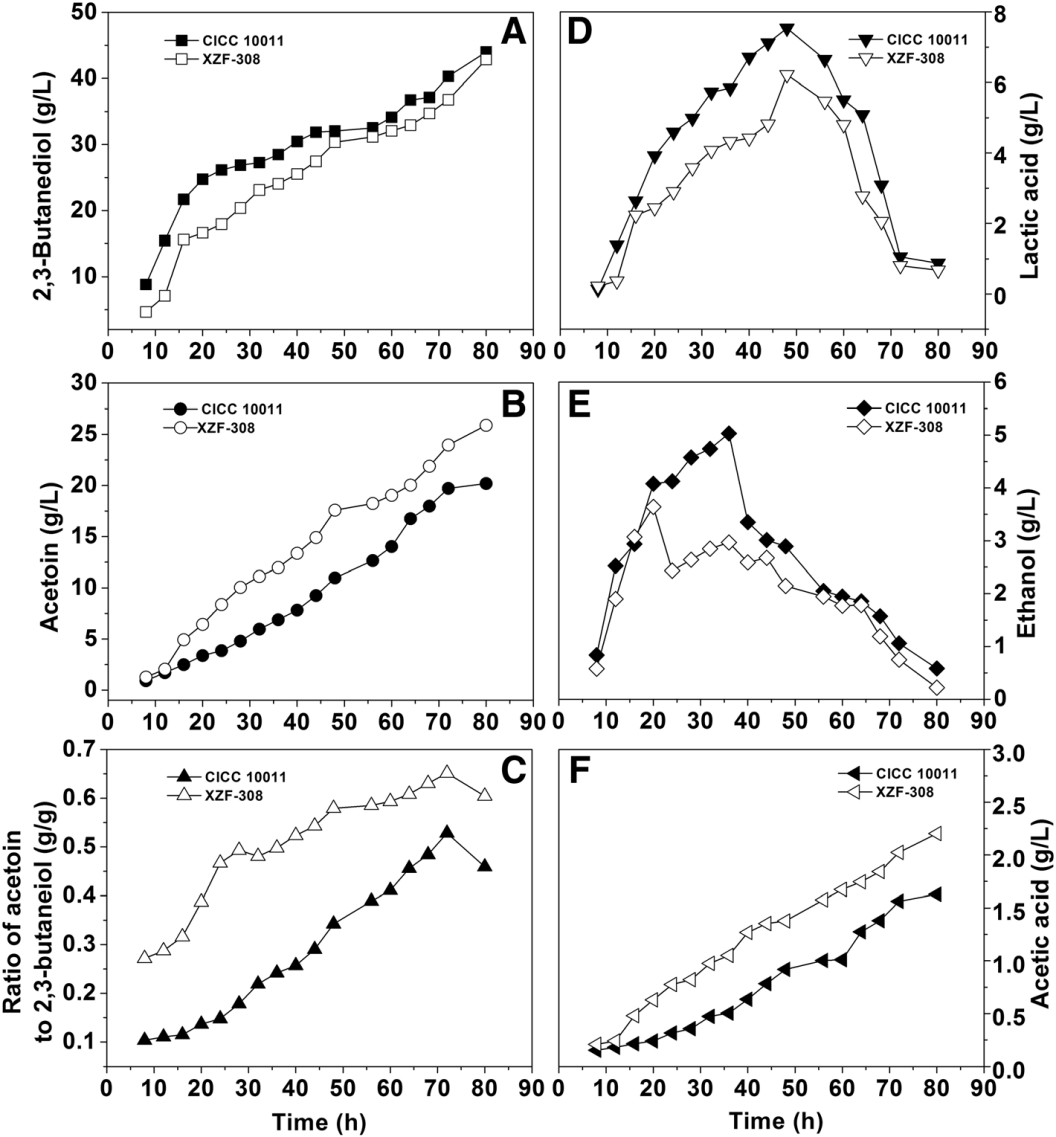
**Effects of the NAD**^**+**^**regeneration system introduced into *Klebsiella pneumoniae *on the metabolite distribution in fed-batch culture.** CICC 10011: the parent strain; XZF-308: the recombinant strain.

As shown in Figure 4B, it was observed that the acetoin titer could not further increase when the glucose was exhausted. Therefore, a fed-batch culture by feeding glucose solution was carried out (Additional file 4: Figure S4). After 80 h, the final acetoin titer reached 25.8 g/L (Figure 5B). While the time courses of the other metabolites had the same tendency with the batch culture (Figure 5A, D, E, F). In the previous studies, *K. pneumoniae* and another *Klebsiella* species-*Klebsiella oxytoca* was used for 2,3-BD production, acetoin which acted as a kind of coupled by-product was collected accompanying with 2,3-BD at the end of fermentation. However, its production titer was generally lower than 15 g/L. Here, the engineered *K. pneumoniae* could accumulate more than 20 g/L acetoin, which is the highest report of acetoin yield by *Klebsiella* species (Table 2).

**Table 2:** **Microbial production of acetoin and 2,3-butanediol using *Klebsiella *species**

**Strains**	**Products (g/L)**		**References**
	**Acetoin**	**2,3-Butanediol**	
*Klebsiella oxytoca*	2.3	118.0	[24]
*Klebsiella oxytoca*	1.9	95.5	[25]
*Klebsiella oxytoca*	1.4	130.0	[8]
*Klebsiella oxytoca*	15.4	86.2	[26]
*Klebsiella oxytoca*	6.4	85.5	[27]
*Klebsiella pneumoniae*	13.1	67.4	[28]
*Klebsiella pneumoniae*	10.0	150.0	[29]
*Klebsiella pneumoniae*	13.1	92.4	[30]
*Klebsiella pneumoniae*	7.6	84.0	[31]
*Klebsiella pneumoniae*	3.6	78.9	[32]
*Klebsiella pneumoniae*	25.9	42.8	This work

## Discussion

In the present study, the heterologous NOX was successfully expressed in *K. pneumoniae* and the effect of introducing NAD^+^ regeneration system on glucose metabolism was investigated. After conducting the cofactor engineering manipulations, the intracellular redox status (showed by the NADH/NAD^+^ ratio) was disturbed. The result was similar to that of engineered *S. cerevisiae*[6,33,34] and *L. lactis*[4], in which the NADH/NAD^+^ ratio was greatly influenced by introducing the system of NAD^+^ regeneration. Owing to changed intracellular redox status, the cell growth of the recombinant *K. pneumoniae* was somewhat inhibited.

The heterologous expression of NOX in *K. pneumoniae* was a useful tool for qualifying the metabolic network and studying the interaction between the NADH level and metabolic fluxes. As indicated in the third part of the ‘Results’ section, the large decrease of the NADH/NAD^+^ ratio due to the introduction of the NAD^+^ regeneration system triggered a dramatic metabolic flux redistribution, as shown by changed concentrations of acetoin, 2,3-BD, acetic acid, lactic acid, and ethanol. Generally, in the mixed acid-2,3-BD fermentation pathway, the synthesis of 2,3-BD requires a large number of NADH along with the reduction of acetoin. Besides, the formation of lactic acid and ethanol also needs NADH and therefore the productivity of these products is related with intracellular NADH and NAD^+^ concentrations. With the decrease of NADH concentration in the recombinant strain, the acetoin production was almost tripled compared to the parent strain in the batch culture (Figure 4B), whereas the 2,3-BD production was nearly the same as the parent strain, which was not anticipated to decrease after the introduction of the NAD^+^ regeneration system. At the same time, in the recombinant, the carbon fluxes to lactic acid and ethanol were decreased due to the fact that the NADH availability was reduced. That is to say, the increase of the acetoin production was at the expense of lactic acid and ethanol production while not 2,3-BD, although accumulation of these metabolites all require NADH.

In fact, in response to the introduction of NAD^+^ regeneration system, the first to be affected were the lactic acid and ethanol metabolic pathways among the three NADH dependent pathways. After introducing the NAD^+^ regeneration system by heterologous expression of NOX, the excess NADH generated by the glycolytic pathway that would be channeled to the respiratory chain to form ATP, was oxidized to H_2_O catalyzed by the NOX. Therefore, the acetic acid pathway was triggered to be enhanced as it could alternatively contribute to generate ATP which was essential for cell growth (Figure 4F and 5F). This could also explain that why the non-NADH dependent acetic acid metabolic pathway was affected by introducing the NAD^+^ regeneration system. Consequently, due to the enhancement of acetic acid formation, the flux of the metabolic pathway of 2,3-BD was triggered to be increased, as acetic acid was proven to be able to act as an inducer for the production of the key enzymes involved in 2,3-BD formation [22,23]. Therefore, driven by the introduced NAD^+^ regeneration system, the increased fluxes to 2,3-BD pathway was reflected by the increased acetoin accumulation with the 2,3-BD formation nearly unaffected.

## Conclusions

In conclusion, the introduction of NAD^+^ regeneration system into *K. pneumoniae* is a powerful engineering tool to enhance the metabolic flux to some desired metabolites. Increasing the availability of intracellular NAD^+^ favored the production of more oxidative metabolites, as evidenced by a significant increase in the acetoin production, while decrease in the production of lactic acid and ethanol. The present study showed that the NOX can be well expressed in *K. pneumoniae,* and heterologous expression of NOX resulted in large decreases in the intracellular NADH concentration and NADH/NAD^+^ ratio, thus altered the metabolite spectrum of the mixed acid-2,3-BD fermentation pathway. To the best of our knowledge, the result in the present study was a new record on acetoin production by *Klebsiella* species. Furthermore, the result in present study provides useful information for increasing accumulation of NAD^+^-dependent microbial metabolite. This is another example showing the advantage of cofactor engineering in terms of manipulating the form and level of intracellular cofactors to redirect cellular metabolism. The idea developed in this paper could be applied to the other similar industrial biotechnological process to achieve high product concentration.

## Materials and methods

### Strains and plasmid construction

The strains, plasmids, and primers used in this study were listed in Table 3. *K. pneumoniae* CICC10011, which was obtained from China Center of Industrial Culture Collection (CICC), was used as the parent strain for acetoin and 2,3-BD production. *E. coli* DH5α was used for plasmid cloning and maintenance. The *nox-2* gene from *S. pneumoniae* encoding a water-forming NADH oxidase was amplified by PCR with Taq polymerase (TaKaRa, Dalian, China) using primers P1 and P2 (primers were based on the sequence of the *nox-2* gene under GenBank accession No. AF014458). The amplified fragment was digested with *Kpn* I and *Pst* I and inserted into a cloning vector pUC18, resulted in pUC18-*nox-2*. The ampicillin-resistant transformants were selected by the blue/white method on plates. White clones were picked out and the plasmid pUC18-*nox-2* was extracted, from which the *nox-2* gene was digested by *Kpn* I and *Pst* I and introduced into the *Kpn* I-*Pst* I site of an expressing vector pDK7 under the control of the tac promoter (Additional file 5: Figure S5). The yielded recombinant plasmid pXZF-308 was transformed into *K. pneumoniae* CICC10011 by standard transformation protocol using the method of electroporation [35-37]. The chloramphenicol-resistant transformants were selected, and the insert was confirmed by colony PCR, restrictive digestion, and sequencing. The confirmed clone harboring pXZF-308 was designated as *K. pneumoniae* XZF-308.

**Table 3:** Bacterial strains, plasmids, and primers used in this study

**Strains, plasmids, or primers**	**Genotypes, properties, or sequences**^**a**^	**Sources or references**
Strains		
*Klebsiella pneumoniae* CICC10011	Ap^r^, parent strain	CICC
*K. pneumoniae* XZF-308	Ap^r^, Cm^r^, recombinant strain of CICC10011, harbring pDK7-*nox*-2	This work
*Escherichia coli* DH5α	supE44 ∆ lacU169 (φ80 lacZ ∆ M15) hsdR17 recA1 endA1 gyrA96 thi-1 relA1	TaKaRa
Plasmids		
pNOX2	pMosBlue carrying a PCR product encoding NADH oxidase of *S. pneumoniae*	[38]
pUC18	Ap^r^, 2686 bp	TaKaRa
pUC18-*nox*-2	Ap^r^, pUC18 derivative carrying a 1380 bp DNA fragment containing the gene of *nox*-2, 4066 bp	This work
pDK7	Expressing vector, Cm^r^, 4800 bp	[39]
pXZF-308	Cm^r^, pDK7 derivative carrying a 1380 bp DNA fragment containing the gene of *nox*-2, 6180 bp	This work
Primers ^b^		
P1	5^′^-CGGGGTACCTAAGGAGGATATACATATGAGTAAAATCGT-3’ (*Kpn* I)	This work
P2	5^′^-CGGCTGCAGTTATTTTTCAGCCGTAAG-3’ (*Pst* I)	This work

^a^ Ap^r^: ampicillin resistance; Cm^r^: chloramphenicol resistance.

^b^ The underlined letters indicate the restriction sites.

### Media and growth conditions

Luria-Bertani (LB) medium was used as the seed culture of *E. coli, K. pneumoniae* and their derivatives. When *K. pneumoniae* was used for electroporation, ethylene diamine tetraacetic acid (EDTA) was added to the LB medium to a final concentration of 0.7 mM [37]. After the electroporation, the seed culture for activating the recombinant *K. pneumoniae* was prepared in the SOC medium [40]. When necessary, ampicillin (60 μg/mL) and/or tetracycline (12 μg/mL) was added to the medium as selection markers.

For seeds preparation, *K. pneumoniae* strains were cultured in a 250-mL Erlenmeyer flask containing 50 mL fresh medium inoculated with a full loop of *K. pneumoniae* from fresh slant tube. The seeds were cultivated at 37°C and 200 rpm on a rotary shaker for 24 h [26]. Seed culture (5%, v/v) was then inoculated into the fermentation medium and batch and fed-batch fermentation were carried out in a 3-L stirred fermenter (BioFlo 100; New Brunswick Scientific Co., NJ, USA) with a an initial broth volume of 2 L. The fermentation medium for *K. pneumoniae* was composed of (g/L): glucose, 100; K_2_HPO_4_, 13.7; KH_2_PO_4_, 2.0; (NH_4_)_2_HPO_4_, 3.3; (NH_4_)_2_SO_4_, 6.6; MgSO_4_·7H_2_O, 0.25; FeSO_4_·7H_2_O, 0.05; ZnSO_4_·7H_2_O, 0.001; MnSO_4_·H_2_O, 0.001; CaCl_2_·2H_2_O, 0.01; EDTA, 0.05 [8]. The fermentation was performed at 37°C with the aeration rate of 1.0 vvm and agitation speed of 400 rpm, respectively. When the pH decreased to 6.5, it was controlled at 6.5 automatically by adding 3 M NaOH. Fed-batch fermentation was conducted by feeding 400 g/L glucose when the residual glucose in the fermentation broth was below 10 g/L.

### Preparation of cell extracts and NOX activity assay

The recombinant strain *K. pneumoniae* XZF-308 was grown in 500-mL Erlenmeyer flasks with 100 mL working volume and also cultured in 3-L fermentor with 2 L working volume. Isopropyl β-d-thiogalactoside (IPTG) was added into the culture to a final concentration of 1 mM to induce the expression of *nox-2* gene when the strains grew to an OD_600_ of 0.6-0.8. After induction at 32°C for 10 h, cell pellets were washed with 35 mM potassium phosphate (pH 8.0) buffer twice, and resuspended in the same buffer. Cells were then disrupted by sonication at 4°C for 10 min. Cell debris was removed by centrifugation at 12,000 rpm and 4°C for 20 min. The supernatant was used for NOX activity assay. According to the previous studies [41], NOX activity was assayed spectrophotometrically at 25°C in a total volume of 1 mL containing 50 mM potassium phosphate (pH 7.0), 0.3 mM EDTA, 10 μM FAD, and 0.29 mM NADH. The reaction was initiated by adding a suitable amount of cell extracts to the reaction mixture and the decrease in absorbance at 340 nm was determined to calculate NADH concentration. A unit of NOX activity was defined as the amount that catalyzed the oxidation of 1 μmol of NADH to NAD^+^ per minute at 25°C. Total protein concentration in cell extracts was determined by the method described by Bradford with bovine serum albumin as standard protein [42].

### Analytical methods

Biomass, which was shown as dry cell weight (DCW, g/L), was determined by measuring the turbidity of the culture at 600 nm using a UV visible spectroscopy system (Lambda-25, Perkin-Elmer, USA). One unit of optical density was determined to be equivalent to 0.35 g DCW per liter. The concentration of acetoin, 2,3-BD, and other by-products (lactic acid, acetic acid, and ethanol) were measured by a high-performance liquid chromatography (Summit P680 HPLC, Dionex, USA; Shodex RI-101 Refractive Index Detector, Showa Denko, Japan; Aminex HPX-87H Ion Exclusion Column 300 mm×7.8 mm, Bio-Rad, USA) under the following conditions: sample volume 10 μL; mobile phase 0.005 M H_2_SO_4_; flow rate 0.6 mL/min; column temperature 60°C.

The intracellular NADH and NAD^+^ concentrations were measured by procedures presented by San et al. [1]. One milliliter culture broth was sampled from the reactor and quickly pipetted into a tube. The samples were then centrifuged at 12,000 rpm for 5 min. The supernatant was removed and 300 μL 0.2 mol/L NaOH for NADH or 300 μL 0.2 mol/L HCl for NAD^+^ was added to the pellets to resuspend them. The NaOH extraction destroyed the oxidized form, and the HCl extraction destroyed the reduced form of pyridine nucleotides [43]. The samples were placed in a water bath at 50°C for 10 min and then transferred to an ice bath for cooling down to 0°C. After neutralization by adding 300 μL 0.1 mol/L HCl for NADH extraction or 300 μL 0.1 mol/L NaOH for NAD^+^ extraction, the samples were centrifuged at 12,000 rpm, 4°C for 10 min. The cell debris was removed, and the supernatant was transferred to a new tube and stored at −20°C. The intracellular NADH or NAD^+^ concentration was determined by the enzymatic cycling assay method [44]. The mixture of cycling asssay consisted of equal volumes of buffer (Bicine buffer, 1.0 mol/L, pH 8.0), absolute ethanol, EDTA (40 mmol/L, pH 8.0), thiazolyl-blue (MTT, 4.2 mmol/L), and twice the volume of phenanzinium ethylsulfate (PES, 16.6 mmol/L), was incubated at 30°C for 10 min. The reaction mixture contained 50 μL neutralized cell extraction, 0.3 mL purified water, 0.6 mL mixture referred above, and 50 μL alcohol dehydrogenase (EC 1.1.1.1; 500 U/mL). The absorbance at 570 nm was checked for 10 min. The concentrations of NADH and NAD^+^ in the supernatant were determined, and the content was calculated by a linear fit equation. This equation was obtained from the slope of the linear region of the absorbance versus time plot, correlated with the concentrations of NADH and NAD^+^.

## Competing interests

The authors declare that they have no competing interests.

## Authors’ contributions

XJJ and HH designed experiments. ZFX, ZKN, NHF, and XJJ performed experiments. MQS and QQT contributed reagents and materials. ZFX and ZKN analyzed data. ZFX, XJJ, and HH wrote the manuscript. All authors have read and approved the final manuscript.

## Supplementary Material

Additional file 1 Metabolic pathways of acetoin and 2,3-butanediol in the bacteria. Click here for file

Additional file 2 **Validation of the expression of NADH oxidase in *Klebsiella pneumoniae* through SDS-PAGE.** Click here for file

Additional file 3 Strategy for feeding glucose during the fed-batch fermentation process. Click here for file

Additional file 4 **The intracellular oxidation-reduction level was affected by expressing heterologous NADH oxidase in *Klebsiella pneumoniae* in the fed-batch fermentation.** Click here for file

Additional file 5 Strategies for constructing the recombinant plasmids. Click here for file
