# Supplementary material for: Cofactor engineering through heterologous expression of an NADH oxidase and its impact on metabolic flux redistribution in Klebsiella pneumoniae
Source: Biotechnol Biofuels. 2013 Jan 25;6:7. doi: 10.1186/1754-6834-6-7 (PMC3563507; doi:10.1186/1754-6834-6-7)
Supplement: Additional file 1 — Metabolic pathways of acetoin and 2,3-butanediol in the bacteria. [file 1754-6834-6-7-S1.pdf]

Additional file 1-Metabolic pathways of acetoin and 2,3-butanediol in the bacteria

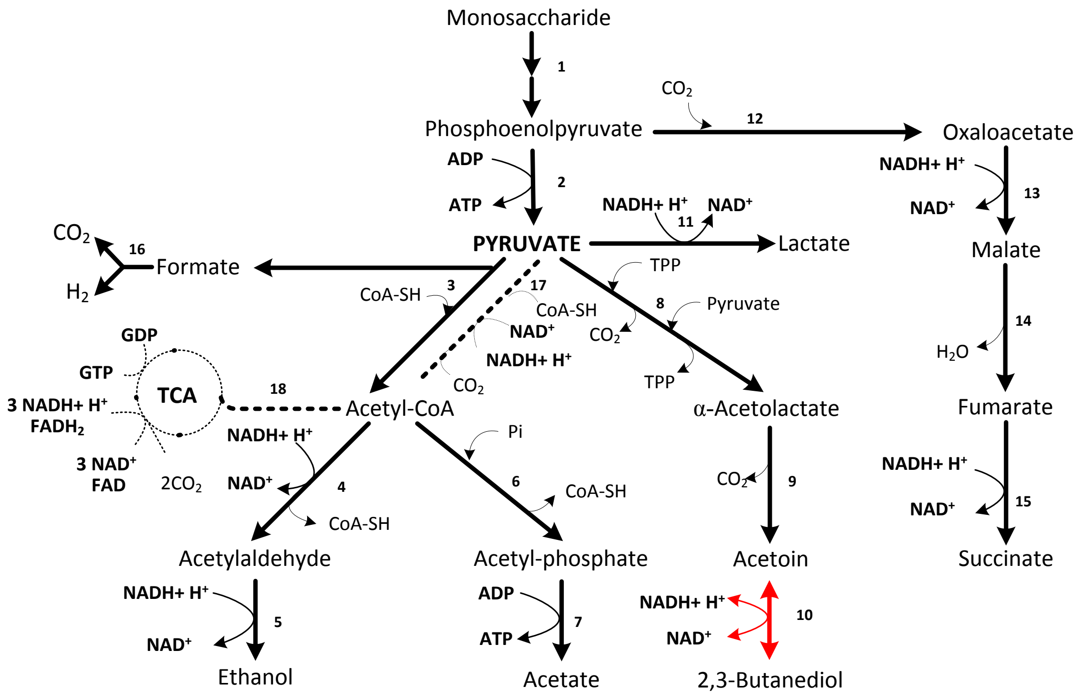

Figure S1. Mixed acid-2,3-butanediol pathway (dashed lines represent the pathways that are active only under the aerobic conditions). TCA: tricarboxylic acids cycle, Enzymes: 1, enzymes of glycolysis (and pentose phosphate pathway); 2, pyruvate kinase; 3, pyruvate-formate lyase; 4, acetaldehyde dehydrogenase; 5, ethanol dehydrogenase; 6, phospho-transacetylase; 7, acetate kinase; 8, acetolactate synthase; 9, acetolactate decarboxylase; 10, acetoin reductase (2,3-butanediol dehydrogenase); 11, lactate dehydrogenase; 12, phosphoenolpyruvate decarboxylase; 13, malate dehydrogenase; 14, fumarase; 15, succinate dehydrogenase; 16, formate-hydrogen lyase; 17, pyruvate dehydrogenase multienzyme complex; and 18, citroyl synthetase.
