# Supplementary material for: Cofactor engineering through heterologous expression of an NADH oxidase and its impact on metabolic flux redistribution in Klebsiella pneumoniae
Source: Biotechnol Biofuels. 2013 Jan 25;6:7. doi: 10.1186/1754-6834-6-7 (PMC3563507; doi:10.1186/1754-6834-6-7)
Supplement: Additional file 2 — Validation of the expression of NADH oxidase in Klebsiella pneumoniae through SDS-PAGE. [file 1754-6834-6-7-S2.pdf]

Additional file 2-Validation of the expression of NADH oxidase in *Klebsiella pneumoniae* through SDS-PAGE

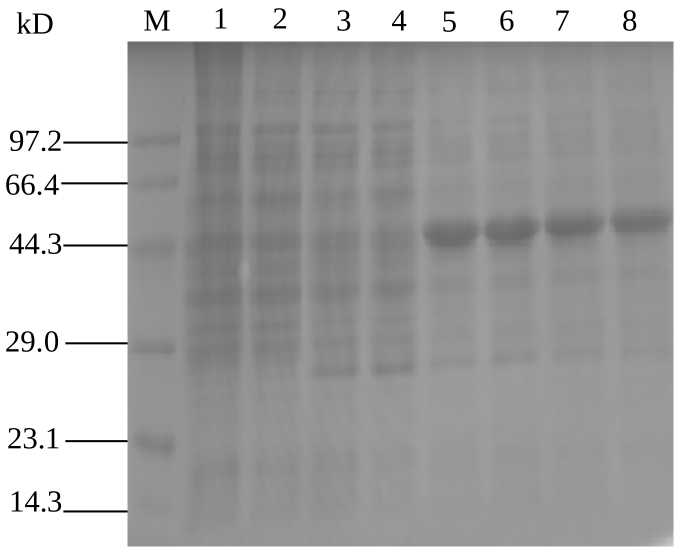

Figure S2. The figure showed the SDS-PAGE analysis of proteins in whole cells of both the parent and recombinant strain at 8 h, 12 h, 16 h, and 20 h, respectively. One protein band about 45 kDa appeared in the recombinant cells, which was in coincidence with the prediction from gene sequence of *nox-2*, therefore, the significant protein band was proved to be NADH oxidase.

M: protein marker; Lane 1: *K. pneumoniae* CICC10011 with IPTG induction at 8 h; Lane 2: *K. pneumoniae* CICC10011 with IPTG induction at 12 h; Lane 3: *K. pneumoniae* CICC10011 with IPTG induction at 16 h; Lane 4: *K. pneumoniae* CICC10011 with IPTG induction at 20 h; Lane 5: *K. pneumoniae* XZF-308 with IPTG induction at 8 h; Lane 6: *K. pneumoniae* XZF-308 with IPTG induction at 12 h; Lane 7: *K. pneumoniae* XZF-308 with IPTG induction at 16 h; Lane 8: *K. pneumoniae* XZF-308 with IPTG induction at 20 h.
