# Supplementary material for: Cofactor engineering through heterologous expression of an NADH oxidase and its impact on metabolic flux redistribution in Klebsiella pneumoniae
Source: Biotechnol Biofuels. 2013 Jan 25;6:7. doi: 10.1186/1754-6834-6-7 (PMC3563507; doi:10.1186/1754-6834-6-7)
Supplement: Additional file 3 — Strategy for feeding glucose during the fed-batch fermentation process. [file 1754-6834-6-7-S3.pdf]

Additional file 3-The intracellular oxidation-reduction level was affected by expressing heterologous NADH oxidase in *Klebsiella pneumoniae* in the fed-batch fermentation

Heterologous expression of NADH oxidase in *K. pneumoniae* led to a lower level of NADH pool and a higher level of NAD^+ pool compared with the parent strain during the fed-batch fermentation process. The difference of the $NADH/NAD^+$ ratio between the parent and the recombinant strain was thus observed.

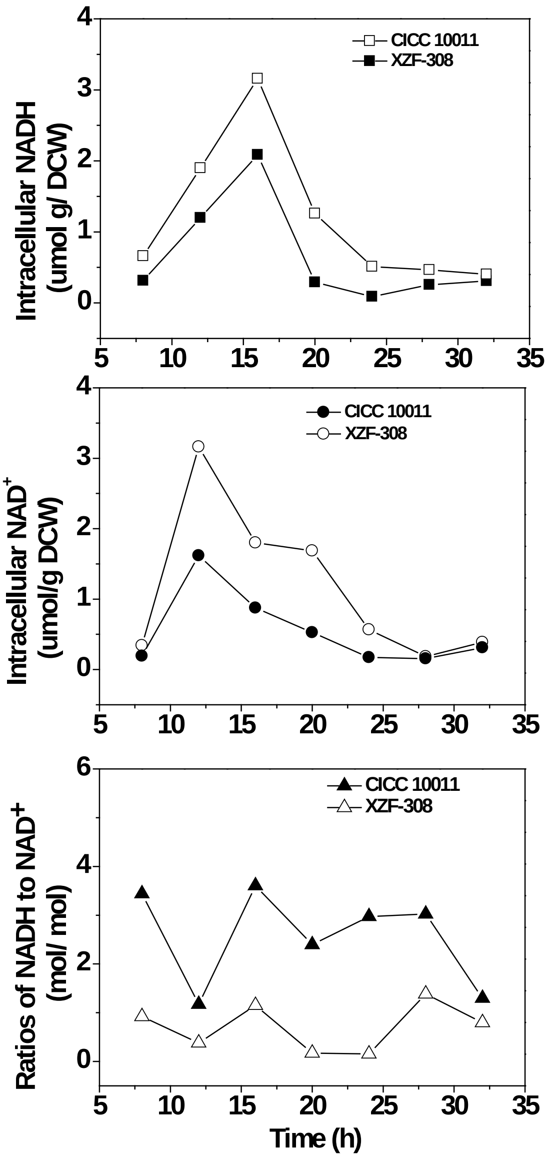

Figure S3. Effects of the NAD^+ regeneration system introduced into *K. pneumoniae* on the concentrations of intracellular NADH, NAD^+, and $NADH/NAD^+$ ratio in fed-batch fermentation. CICC 10011: the parent strain; XZF-308: the recombinant strain.
