# Supplementary material for: Cofactor engineering through heterologous expression of an NADH oxidase and its impact on metabolic flux redistribution in Klebsiella pneumoniae
Source: Biotechnol Biofuels. 2013 Jan 25;6:7. doi: 10.1186/1754-6834-6-7 (PMC3563507; doi:10.1186/1754-6834-6-7)
Supplement: Additional file 4 — The intracellular oxidation-reduction level was affected by expressing heterologous NADH oxidase in Klebsiella pneumoniae in the fed-batch fermentation. [file 1754-6834-6-7-S4.pdf]

Additional file 4-Strategy for feeding glucose during the fed-batch fermentation process

Fed-batch fermentation was conducted by feeding 400 g/L glucose when the residual glucose in the medium was below 10 g/L. Samples were collected periodically to determine the residual glucose concentration, and the rate of feeding glucose was adjusted to keep the glucose concentration at a comparatively low level.

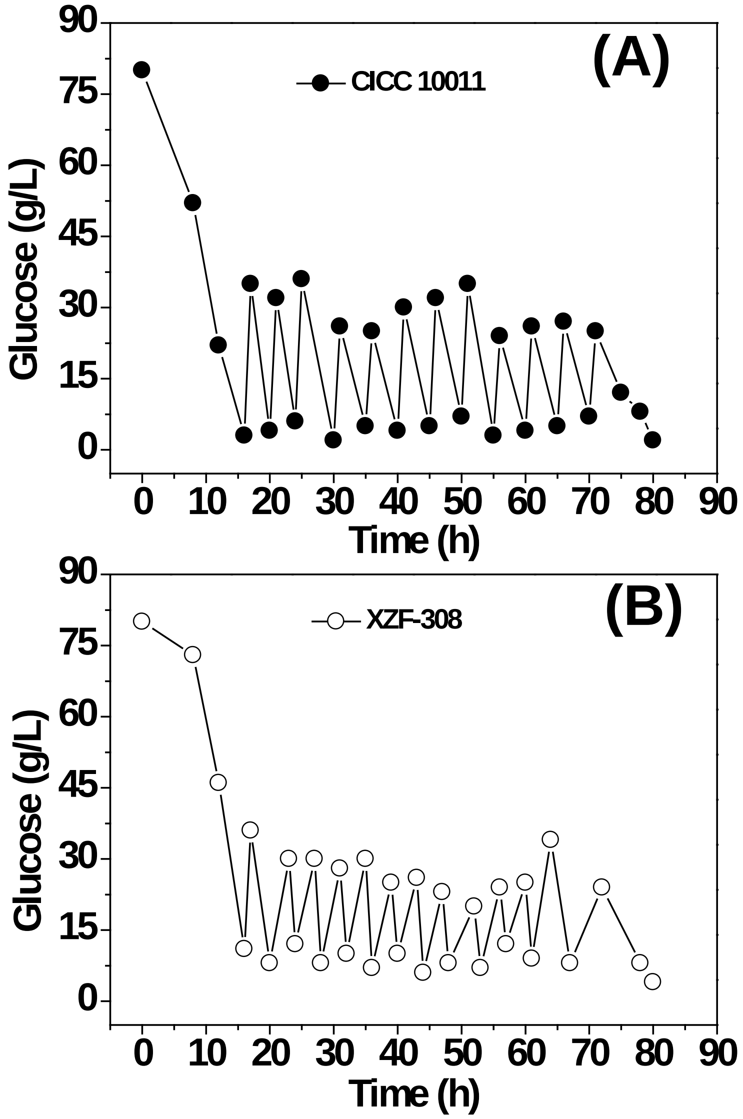

Figure S4. Time course of glucose consumption in the fed-batch culture of *Klebsiella pneumoniae*. CICC 10011: the parent strain; XZF-308: the recombinant strain.
