# Supplementary material for: Cofactor engineering through heterologous expression of an NADH oxidase and its impact on metabolic flux redistribution in Klebsiella pneumoniae
Source: Biotechnol Biofuels. 2013 Jan 25;6:7. doi: 10.1186/1754-6834-6-7 (PMC3563507; doi:10.1186/1754-6834-6-7)
Supplement: Additional file 5 — Strategies for constructing the recombinant plasmids. [file 1754-6834-6-7-S5.pdf]

Additional file 5-Strategies for constructing the recombinant plasmids.

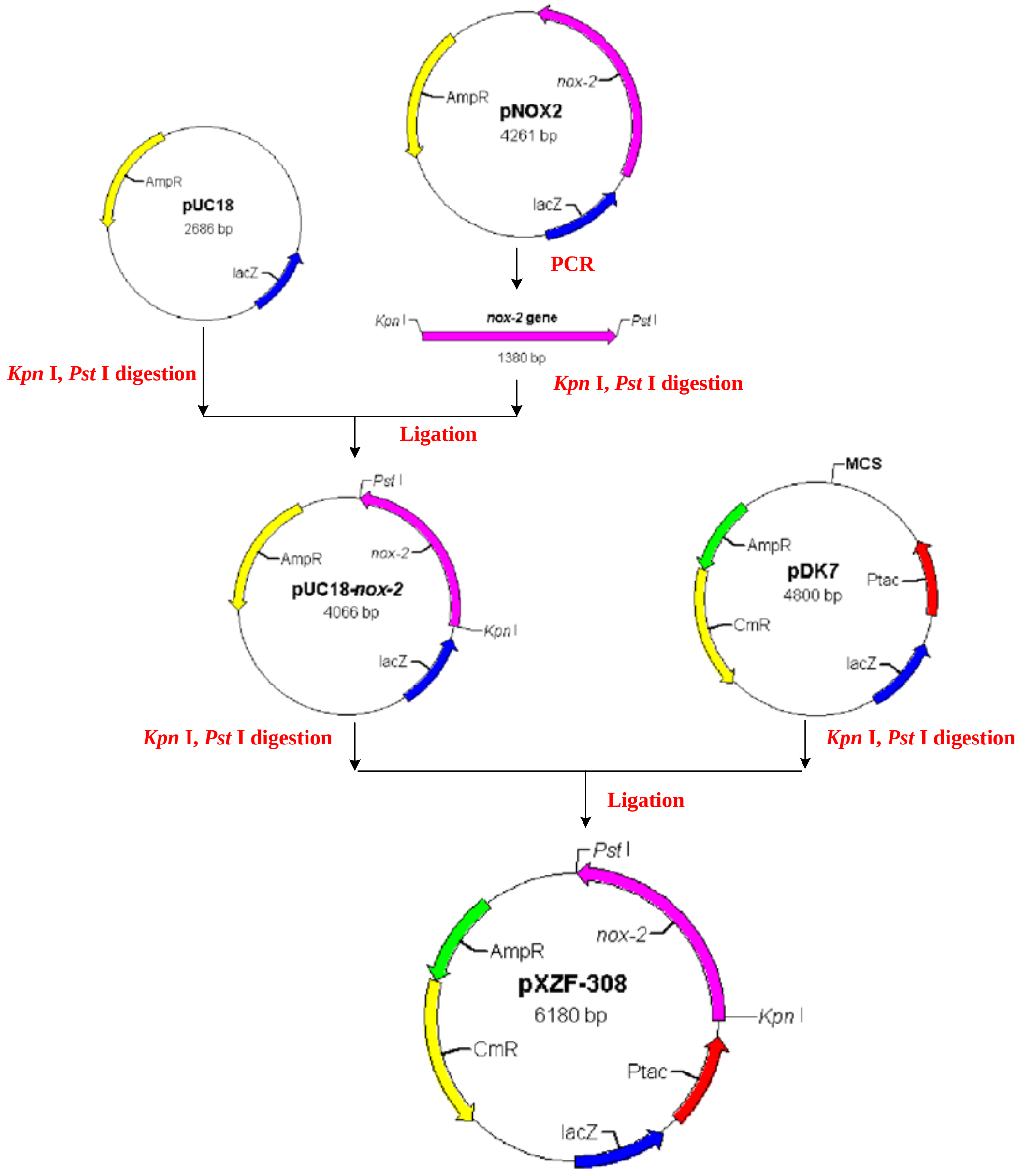

Figure S5. Strategies for constructing the recombinant plasmids of pUC18-nox-2 and pXZF-308. The *nox-2* gene from *S. pneumoniae* encoding a water-forming NADH oxidase was amplified from pNOX2 by polymerase chain reaction. The *nox-2* gene fragment was then inserted into the restriction sites of *Kpn* I and *Pst* I in pUC18, yielding the recombinant plasmid pUC18-nox-2. Then, the *nox-2* gene fragment was digested by *Kpn* I and *Pst* I, and inserted into the *Kpn* I-*Pst* I site of an expressing vector pDK7, yielding the recombinant strain pXZF-308.
